# Is England facing an opioid epidemic?

**DOI:** 10.1177/20494637231160684

**Published:** 2023-02-27

**Authors:** Antonia-Olivia Roberts, Georgia C Richards

**Affiliations:** 1 Medical Sciences Division, 6396 University of Oxford, Oxford, UK; 2 Green Templeton College, 6396 University of Oxford, Oxford, UK; 3 Centre for Evidence-Based Medicine, Nuffield Department of Primary Care Health Sciences, 6396 University of Oxford, Oxford, UK

**Keywords:** Chronic pain, pain clinics, pain management, pain, low back pain

## Abstract

The opioid crisis in the United States (US) is one of the most high-profile public health scandals of the 21st century with millions of people unknowingly becoming dependent on opioids. The United Kingdom (UK) had the world’s highest rate of opioid consumption in 2019, and opiate-related drug poisoning deaths have increased by 388% since 1993 in England and Wales. This article explores the epidemiological definitions of public health emergencies and epidemics in the context of opioid use, misuse, and mortality in England, to establish whether England is facing an opioid crisis.

The opioid crisis in the United States (US) is one of the most high-profile public health scandals of the 21^st^ century with millions of people unknowingly becoming dependent on opioids. The United Kingdom (UK) had the world’s highest rate of opioid consumption in 2019,^1^ and opiate-related drug poisoning deaths have increased by 388% since 1993 in England and Wales.^2^ This article explores the epidemiological definitions of public health emergencies and epidemics in the context of opioid use, misuse, and mortality in England, to establish whether England is facing an opioid crisis.

## Definitions

Definitions applied to public health problems have extensive economic and social impact. The World Health Organization (WHO) received backlash after declaring the spread of H1N1, a pandemic in 2009; a virus that caused fewer deaths than the average flu season.^3–5^ This was because their criteria for defining pandemics were based on the geographic distribution of cases and lacked the inclusion of morbidity or mortality.^6^ Post-2009, the criteria for pandemics have been abandoned in favour of a case-by-case approach.^7^ The United States (US) Centre for Disease Control and Prevention (CDC) defines an epidemic as ‘the occurrence of more cases of disease, injury or other health condition than expected in a given area or among a specific group of persons during a particular period’.^8^ The Dictionary of Epidemiology defines an epidemic as ‘the occurrence in a community or region of cases of an illness… clearly in excess of normal expectancy’.^9^ The overdose and death of more than 932,000 people from opioids since 1999^10^ in the US was deemed a ‘public health emergency’, a term not defined by the WHO or CDC.

## The US public health emergency

On 26 October 2017, the then US President, Donald Trump, and the Department of Health and Human Sciences (HHS) declared the increasing number of opioid overdose deaths a ‘public health emergency’.^11^ The HHS defined a public health emergency as the ‘need for health care services to respond to a disaster… or other significant or catastrophic event’.^12^ This definition also does not include the extent of morbidity or mortality. Public health experts have established a definition for public health emergencies which incorporates health consequences, their causes, and precipitating events, where the ‘scale, timing, or unpredictability threatens to overwhelm routine capabilities’.^13^ Trump resisted pressure from the Presidential Opioid Commission to declare a more serious ‘national emergency’, which could allow emergency relief funds to be used.^14,15^ Therefore, the decision of the US to declare a public health emergency rather than a national epidemic may have been influenced by political and financial motivations.

There were three distinct hallmarks of the US opioid crisis; the widespread increase in opioid prescribing with subsequent opioid dependence and addiction; increased use of street opioids, including heroin; and subsequently, the introduction of highly potent synthetic opioids such as fentanyl.

### Part one: Opioid prescribing and deaths

The crisis in the US began in the mid-1990s with the excessive prescribing of opioids, in part due to aggressive marketing tactics by pharmaceutical companies^16^ and small, poorly conducted trials and case reports assessing opioid efficacy.^17–19^ The widespread prescribing of opioids in the US led to a death rate of 20.7 per 100,000 people in 2020.^20^ In England, the opioid death rate has been substantially lower at 4.0 per 100,000 people in 2020, yet the prescribing of high-dose and long-acting opioids increased by 457% from 1998 to 2018.^21,22^ The differences in death rates may be driven by the contrasting healthcare systems. The UK’s National Health Service (NHS) provides universal access to healthcare and has a centralised model of primary care services whereby patients must register with a single general practice. In the US, the physician network is decentralised, enabling patients to ‘doctor shop’ to collect multiple opioid prescriptions. Furthermore, the US has direct-to-consumer marketing of pharmaceuticals, which increases demand and knowledge of opioids as a ‘quick fix’ for all pain conditions.

Although the opioid death rate is lower in England than the US, England is still experiencing higher than expected rates of mortality and morbidity from opioids. Opioid-related hospitalisations increased by 49% from 2008 to 2018,^23^ and the number of people receiving treatment for prescription and over-the-counter (OTC) drug misuse also increased between 2009 and 2016.^24^ Factors that contributed to the increased opioid mortality and morbidity in England may include the socio-economic fallout from the 2008 financial crisis, higher unemployment rates and deprivation, and the increasing ageing population that puts more people at risk of developing chronic non-cancer pain.^22,25,26^ However, mortality statistics in England and Wales do not distinguish between the source of opioid involved in drug poisonings (i.e., prescribed, illicit, or a combination). Although, the number of methadone-related deaths significantly increased in England in 2021,^2^ and methadone is mainly used in opioid substitution therapy to treat misuse, suggesting that part of the increase is due to the illicit use of opioids.^2^ Whilst the volume of opioids prescribed in the NHS has declined modestly since 2017,^22^ the outcomes of this reduction remain unclear.

### Part two: Street heroin

The second stage of the US opioid crisis began in 2010 with the move from prescribed to street drugs, such as heroin.^16^ In the US, an analysis of National Survey on Drug Use and Health data showed that recent heroin use was 19 times higher amongst those with prior non-medical use of pain medicines.^27^ In the US, 0.2% of the population over 12 years old had a heroin use disorder,^28^ and in 2020, there were 3.97 heroin overdose deaths per 100,000.^29^ In England, the heroin and morphine death rate in 2020 was 2.34 per 100,000, which has risen by 667% since 1993.^2^ However, in England, statistics on heroin deaths are not separated from morphine-related deaths, and it is not possible to determine whether they were from prescribed or illicit sources, or a combination, as both drugs are available as prescriptions (e.g. diamorphine; however, only a small number of prescriptions of diamorphine are dispensed as they require a license from the Home Office).^30,31^ Between 2010 and 2012, there was a decrease in the number of deaths from heroin and morphine because of a ‘heroin drought’ in England, which reduced the supply.^32^ However, since 2013 the supply of heroin has increased in England, which may be driving the increased mortality.^32^ Other factors that may be increasing the mortality may include the ageing of heroin users who commenced heroin during the heroin ‘epidemic’ of the 1980s–1990s.^33^ Heroin usage confers complex health problems and added vulnerability to overdose.^32^

### Part three: Synthetic opioids (fentanyls)

The third wave of the US opioid crisis started in 2013 as usage and deaths related to synthetic opioids, primarily fentanyl, increased.^16^ Synthetic opioids are more potent than naturally occurring opioids; fentanyl is 50–100 times stronger than morphine.^34^ In the US, the death rate for synthetic opioids excluding methadone was 21.4 per 100,000 in 2021.^35^ In comparison, the fentanyl death rate in England was 0.223 per 100,000 in 2021. One of the predictive factors for rates of fentanyl overdose in the US was the rate of opioid prescribing.^36^ Whilst fentanyl-related deaths remained fewer than 10 per year from 1998 to 2009 in England, they reached 79 deaths in 2017.^37^ Similar increases were found for fentanyl analogue-related deaths, from fewer than 5 deaths per year until 2016 to 51 deaths in 2017.^37^ However, it is not possible to determine whether these opioids were prescribed, illicit, or both. Despite the rate of synthetic opioid-related deaths in England being less than the US, the recent increase is concerning and requires close monitoring and the provision of services.^32^

## Access to health services in England

A key factor in determining whether a public health emergency has occurred is the extent to which health services are impacted. In England, the number of people in treatment for problems with prescription and OTC medicines has increased by 38%, from 4800 people in 2009 to 6600 people in 2016.^24^ However, the proportion of people using opioids who were in treatment has decreased from a peak of 65% in 2008–2009 to 54% in 2019–2020.^38^ Although, fewer opioid users are completing treatment successfully, and a higher proportion of people in treatment for opioid use are dying.^38,24^ Concerningly, the funding for addiction services has also decreased, which has resulted in high caseload numbers for staff in treatment services.^38^ Access to chronic pain services also remains inadequate in England and Wales.^39^ A National Pain Audit of specialist pain services in England and Wales between 2010 and 2014 found that services fell below the recommended standards of care, with few clinics achieving evidence-based recommended waiting times and an availability of multidisciplinary staffing.^40^ Therefore, it appears that access to addiction and chronic pain services are inadequate in England.

## Chronic non-cancer pain

Opioids have been commonly prescribed for people with chronic non-cancer pain. In the UK, a cross-sectional study in primary care found that 88% of strong opioids were prescribed to people for non-cancer pain between 2000 and 2010.^43^ In the US, an analysis of commercial and public health insurance data found that 51% of people prescribed opioids had non-cancer pain.^44^ However, the prevalence of chronic pain in the US is reported to be lower than the UK. A systematic review of population studies found that between one-third and one-half of the adult population in the UK reported chronic pain,^41^ whereas one in five was reported in the US.^42^ However, definitions for chronic pain and data collection methods can vary.

Clinical guidelines for the management of chronic primary pain were published by the National Institute for Health and Care Excellence (NICE) in April 2021, which advise against the use of opioids.^45^ These guidelines were the first to recognise chronic primary pain as a condition in its own right. However, since the introduction of the guidelines, 47% of people with chronic pain still report the use of opioids.^46^ One of the drivers of prescribing opioids for chronic pain is that the preferred treatments are non-pharmacological interventions, including exercise and cognitive behavioural therapy (CBT),^45^ which are difficult to ensure engagement and harder to access for people in deprived areas in England.

## Conclusions

England has experienced a dramatic increase in the prescribing of opioids concurrent with a rise in opioid-related deaths and the number of people seeking treatment for prescription opioid misuse. Whilst opioid mortality rates in England have not reached the levels of the US, the harms of opioid use and mortality have continued to increase. Therefore, the situation in England can be defined as an opioid epidemic but not a public health emergency like the US, as opioid addiction, overdose, and deaths have not yet threatened to overwhelm routine health services. However, it appears that England is facing a chronic pain emergency where investment and access to pain services for people with chronic pain and addiction are urgently needed.
